# The Mechanism of *Microbial-Ferromanganese Nodule Interaction* and the Contribution of Biomineralization to the Formation of Oceanic Ferromanganese Nodules

**DOI:** 10.3390/microorganisms9061247

**Published:** 2021-06-08

**Authors:** Jing Lyu, Xinke Yu, Mingyu Jiang, Wenrui Cao, Gaowa Saren, Fengming Chang

**Affiliations:** 1 CAS Key Laboratory of Marine Geology and Environment, Institute of Oceanology, Chinese Academy of Sciences, Qingdao 266071, China; jinglyu@ufl.edu (J.L.); xyu@qdio.ac.cn (X.Y.); wrcao@qdio.ac.cn (W.C.); saren@qdio.ac.cn (G.S.); chfm@qdio.ac.cn (F.C.); 2 Department of Geological Sciences, University of Florida, Gainesville, FL 32611, USA; 3 Center for Ocean Mega-Science, Chinese Academy of Sciences, Qingdao 266071, China

**Keywords:** ferromanganese nodules, microorganisms, biomineralization

## Abstract

Ferromanganese nodules are an important mineral resource in the seafloor; however, the genetic mechanism is still unknown. The biomineralization of microorganisms appears to promote ferromanganese nodule formation. To investigate the possible mechanism of microbial–ferromanganese nodule interaction, to test the possibility of marine microorganisms as deposition template for ferromanganese nodules minerals, the interactions between *Jeotgalibacillus campisalis* strain CW126-A03 and ferromanganese nodules were studied. The results showed that strain CW126-A03 increased ion concentrations of Fe, Mn, and other metal elements in solutions at first. Then, metal ions were accumulated on the cells’ surface and formed ultra-micro sized mineral particles, even crystalline minerals. Strain CW126-A03 appeared to release major elements in ferromanganese nodules, and the cell surface may be a nucleation site for mineral precipitation. This finding highlights the potentially important role of biologically induced mineralization (BIM) in ferromanganese nodule formation. This BIM hypothesis provides another perspective for understanding ferromanganese nodules’ genetic mechanism, indicating the potential of microorganisms in nodule formation.

## 1. Introduction

Marine ferromanganese nodules represent an important mineral resource on the seafloor, containing a wide variety of elements, such as Fe, Mn, Cu, Co, Ni, Mo, and Li. Ferromanganese nodules are spherical and irregular in size (1–15 cm) and typically distributed in the abyssal plains with water depths of 4000–6000 m [1]. The manganese minerals in nodules are usually composed of barium magnesia manganese ore, hydro manganese ore, and sodium manganese ore. Iron minerals are mainly composed of goethite and hematite [2]. The reserves of ferromanganese nodules are enormous, more than 3 trillion tons, with high economic value. Ferromanganese nodules, together with ferromanganese crusts, are evaluated as potential metal resources [3]. There are many previous types of research on ferromanganese nodules.

The confounding issue is that the ferromanganese nodules’ genetic mechanism is complex. The formation of ferromanganese nodules was once considered as an abiotic process, which may be influenced by many factors, such as submarine tension and fluid activity, tectonic movement, submarine current changes, carbonate compensation depth (CCD), terrigenous clastic supply rate, and marine primary productivity [4]. It was believed that ferromanganese nodules were formed by the precipitation of trace elements in seawater under the control of physiochemistry, colloidal chemistry, and biochemistry. The colloids formed by adsorbing trace elements precipitate on the seabed bedrock in the form of hard crusts of amorphous oxides or hydroxides [5]. According to the genetic mechanism, ferromanganese nodules can be divided into hydrogenetic type, diagenetic type, and mixed type. Hydrogenetic nodules obtain all elements from marine bottom water, while diagenetic nodules obtain elements from the redox cycle in the early diagenetic reaction of organic matter in sediments. The transition from the diagenetic process to the hydrogenetic process depends on the redox conditions in the environment. The mixed-type nodules are composed of different proportions of diagenetic and hydrogenetic end-member laminae [6]. However, Thiel [7] published the first comprehensive report on the potential involvement of microorganisms in the precipitation of manganese nodules and suggested that microorganisms may promote the deposition of Mn in the marine environment. Previous work revealed biofilms and filamentous microorganisms related to the surface of nodules by using scanning electron microscopy (SEM). Recent findings have proposed a microbial-mediated mechanism for nodules formation because X-ray and microscopy studies have shown that the bacterial concentration in manganese-rich nodules is very high [8]. Microorganisms control the dissolution or formation of minerals by catalysis or other ways [9]. Minerals and even rocks, generally formed at higher geological temperatures and pressures, can be formed in the surficial environment through microbial-mediated biochemical and biophysical processes [10]. Due to their small size and diverse metabolic capability, bacteria interact more efficiently with metal ions in the environment than any other type of organism [11]. Previous studies have found that bacteria accumulated metal ions and incorporated ions into mineral phases to promote mineral formation [12,13,14,15]. This process is termed biomineralization. Biomineralization can be divided into biologically induced mineralization (BIM) and biologically controlled mineralization (BCM). BIM occurs outside of the microorganism. Microorganisms’ activities change the surrounding microenvironment, resulting in pH and redox condition changes, then promote extracellular mineral precipitation [16]. In the process of BIM, microorganisms have no apparent control over the formation of minerals, whereas, in BCM, microorganisms directly control the formation of minerals and even control the structure and arrangement of minerals. For example, magnetotactic bacteria form minerals by BCM [17,18]. Hassan et al. found the biological magnetite, which may be formed by magnetotactic bacteria in ferromanganese nodules [19]. Fe-oxidizing bacteria (FeOB) and Mn-oxidizing bacteria (MnOB) may promote the ferromanganese nodules formation by BCM. Phylogenetically diverse MnOB have been reported, including *Bacillus*, *Pseudomonas*, *Leptothrix*, *Erythrobacter*, *Pedomicrobium*, and *Roseobacter*, with enzymatic or superoxide-mediated reactions [20]. MnOB participates in the marine manganese cycle [21,22,23], oxidizing Mn^2+^ to high valent manganese oxides and appear to promote the formation of deep-sea ferromanganese nodules [24]. Microorganisms also affect the migration, accumulation, transformation, and precipitation of Fe-bearing minerals in nature [25,26]. These studies imply the possibility of biomineralization during the formation of ferromanganese nodules.

Little is known about the physiology and metabolism of microorganisms associated with ferromanganese nodules in the ocean and the possible impact of these microbial processes on global marine metal chemistry. Based on the 16S rRNA gene sequence analysis of the microbial community of ferromanganese nodules and the surrounding sediments, recent studies found that the microbial community of nodules was significantly different from that of the surrounding sediments, and the number of bacteria and archaea in nodules was higher than that in the surrounding sediments [27,28]. In the bacterial community composition of ferromanganese nodules [8,20,27,29,30], proteobacteria were dominant, represented by Gammaproteobacteria. Relatives of Gammaproteobacteria, such as *Pseudomonas putida* GB-1, contain two kinds of copper oxidase, which have the potential to oxidize Mn (II) and Mn (III). Detailed phylogenetic analysis of dominant operational taxonomic units (OTUs) associated with Gammaproteobacteria placed them in *Shewanella* and *Colwellia* genera. *Shewanella* strains can reduce Mn under low oxygen conditions, and the oxidized Mn acts as a terminal electron acceptor. No Mn-cycling bacteria such as *Shewanella* and *Colwellia* were detected in the sediments surrounding the nodules. Other phylogenetic groups, such as Nitrospira, Bacteriodetes, Actinobacteria, Acidobacteria, and Alpha-, Beta-, and Deltaproteobacteria, were also detected. The diversity of the Archaea community is less than that of bacteria. Almost all of the obtained archaeal clone sequences from both the ferromanganese nodule and surrounding sediment can be assigned to the Marine Group I (MGI) Thaumarchaeota. Nayak et al. observed the bacterial fossils in ferromanganese nodules by SEM [31]. It was found that the contents of Mn, Ni, and Co around these bacterial fossils were relatively high, which indicated that the bacteria played a particular role in the accumulation of Mn and specific trace elements in these ferromanganese nodules. Jiang et al. found abundant microfossils and biomarkers in ferromanganese nodules and crusts in the South China Sea, and the areas rich in microfossils contain higher Mn than other areas [32]. They suggested that microorganisms may be used as templates to induce mineral deposition in nodules and crusts. However, there is still no solid evidence to explain how microorganisms contribute to the formation of ferromanganese nodules. Therefore, it is meaningful to study the *Microbial-Ferromanganese Nodule Interaction* for understanding the formation process of ferromanganese nodules.

*Jeotgalibacillus campisalis* strain CW126-A03 was selected, and the species *Jeotgalibacillus campisalis* of the family *Planococcaceae* in the phylum *Firmicutes* was first established and combined by Yoon et al. [33,34]. This study aims to explore the role of bacterial biological characteristics and BIM in the formation of ferromanganese nodules. The selected experimental strain is the non-ferromanganese nodule specific and non-MnOB bacterium. The microbial-ferromanganese nodule interaction experiments tested the possibility of microorganisms as the nucleation site of nodules minerals. In this study, a variety of analytical techniques, including X-ray fluorescence (XRF), inductively coupled plasma optical emission spectrometry (ICP-OES), inductively coupled plasma mass spectrometry (ICP-MS), transmission electron microscopy (TEM) with selected area electron diffraction (SAED) and X-ray diffraction (XRD) were utilized to investigate the detail process.

## 2. Materials and Methods

### 2.1. Microorganism

A 50 × 50 cm^2^ square-corer core was obtained from the South China Sea (5°36.51′ N, 113°38.97′; depth: 2125 m). The sampled sediment surface temperature was 9 °C, the pH was 7.4, and the Eh was 201. All the cores were opened from the side onboard, and sub-samples were collected from the center of the core under sterile conditions for microbial study. Then, they were stored in airtight sterile plastic bags at −80 °C freezer until the commencement of laboratory work. Serial dilutions (1:10) of the samples were plated on an artificial seawater medium (yeast extract 1 g/L, peptone 5 g/L, and agar 15 g/L) at 25 °C. The composition of artificial seawater was as follows: 24.32 g/L NaCl, 10.98 g/L MgCl_2_·6H_2_O, 4.06 g/L Na_2_SO_4_, 0.20 g/L NaHCO_3_, 0.027 g/L H_3_BO_3_, 0.10 g/L KBr, 0.69 g/L KCl, 1.14 g/L CaCl_2_. One isolate, which we designated as CW126-A03, was selected for further characterization.

The 16S rRNA gene was amplified by the universal primers 27F and 1492R as previously described [35]. The PCR product was purified and ligated into the PMD19-T vector (Takara) and cloned using the manufacturer’s instructions. Sequencing was performed by BGI (Qingdao, China). The GenBank/EMBL/DDBJ accession number for the 16S rRNA gene sequences of strain CW126-A03 is MT845653.

Strain CW126-A03 was grown by shaking in the sterile artificial seawater medium (yeast extract 1 g/L and peptone 5 g/L) at 25 °C for harvesting the cell biomass. The strain was precultured for 24 h in the 300 mL sterile growth medium, and 10 mL growing cell preculture was then transferred to a conical flask with 300 mL sterile artificial seawater medium. After 24 h of culture, the cells were harvested by centrifugation for 10 min at 4000 r/min. The cells were washed 3 times with 0.1 mol/L NaCl solution to eliminate the growth medium and then resuspended in sterile artificial seawater solution used for the microbial-ferromanganese nodule interaction experiments (hereinafter referred to as interaction experiments). All media were prepared in ultrapure water, and the sterilization condition was at 120 °C for 30 min.

### 2.2. Ferromanganese Nodules

**Figure 1:**
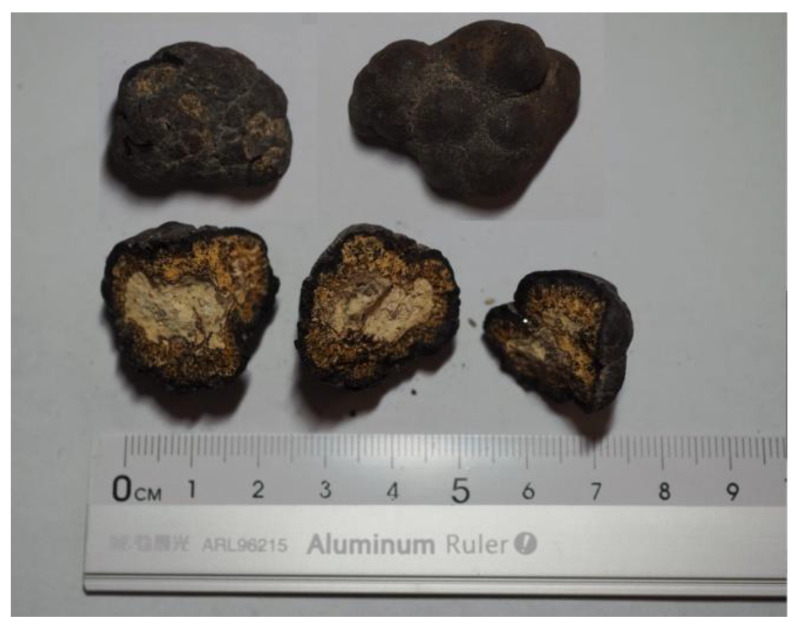
Morphology of ferromanganese nodule samples.

Ferromanganese nodule samples were collected from the East Philippine Sea in the Western Pacific Ocean by the Research Vessel *KEXUE* Expedition 2014, with 4092 m. Ferromanganese nodules were about 3 cm in diameter size, with black spherical and tumorous appearance. The profile feature was the outer black crust wrapping the inner sediments (Figure 1). The outer black crust was peeled off and ground in an agate bowl, which was sieved to isolate the 250 μm size powder as experimental materials. The sieved powder was ultrasonicated by ultrapure water and dried at 60 °C. Then it was sterilized at 120 °C for 30 min.

### 2.3. Microbial-Ferromanganese Nodule Interaction Experiments

All interaction experiments were performed in conical flasks containing 300 mL sterile artificial seawater solution. Experiments were performed twice under the same condition, and the average data from the parallel experiment were shown. Bacterial groups and no bacterial control groups were designed. For bacterial groups, the cells harvested by centrifugation were added to the conical flasks containing sterilized artificial seawater solution, the cell density was determined by spectrophotometer (OD600), and 3 g sterilized Fe-Mn crust powder was added to the conical flasks. For control groups, 3 g sterilized crust powder was added to the conical flasks containing sterile artificial seawater solution. Both groups were performed for 21 days at 120 rpm and 25 °C in the shaking incubator. Samples were taken at different times within 21 days to determine the ion concentration changes in the reaction solution.

### 2.4. Analytical and Statistical Methods

The growth curve of strain CW126-A03 was drawn by measuring *OD*_600_ with a Spectrophotometer (Shanghai Metash Instruments Co., Ltd., UV-5500PC, Shanghai, China). The elements in ferromanganese nodule samples were determined by XRF (Bruker Company, SB Tiger Wavelength Type, Bremen, Germany).

The ion concentrations of Fe, Mn, Co, Ni, and Cu in the reaction solution were measured by ICP-OES (PerkinElmer, Optima 7300DV, Waltham, MA, USA) and ICP-MS (Thermo Fisher, ICAP-QC, Waltham, MA, USA). The analytical samples were prepared by filtering a reaction solution through a 0.22 μm PTFE membrane. Each analytical sample was measured three times, and the relative standard deviations of Fe, Mn, Co, Ni, and Cu were about 3%, 1%, 1%, 2%, and 2%, respectively. The Wilcoxon signed-rank test was used to classify any significant difference between bacterial and control groups.

The morphology of strain CW126-A03 and the minerals formed on the cells’ surface were observed by TEM (Hitachi, HT7700, Tokyo, Japan). The reaction solution was taken out to centrifuge for 10 min at 4000 rpm and washed 3 times with ultrapure water. After that, a small amount of solution was dropped onto the copper mesh and dried at room temperature, then observed by TEM.

The mineral composition changes of ferromanganese nodules were analyzed by XRD (Bruker D-8, Bremen, Germany). The crust powder in solution was collected by centrifugation for 10 min at 4000 rpm and dried at 60 °C.

All statistical analyses were done with R statistical software and Microsoft Excel.

## 3. Results

### 3.1. Growth Curve

**Figure 2:**
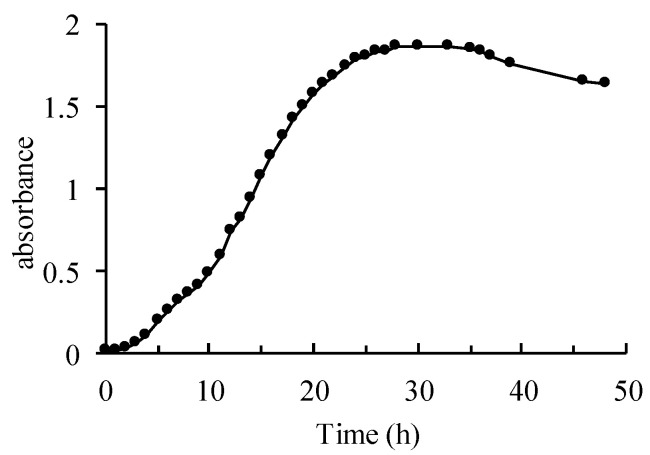
Growth curve of strain CW126-A03.

Strain CW126-A03 was grown by shaking in the incubator at 120 rpm under 25 °C. The growth curve of strain CW126-A03 was in the lag phase for the first 2 h, where bacteria are metabolically active. From 2–24 h, the growth curve was in the log phase, an exponential growth period of bacteria. After 24 h, the growth curve was in the stationary phase, and the number of dying cells equals the number of dividing cells. Finally, the growth curve was in the death phase, and the number of living cells began to decrease (Figure 2).

### 3.2. Elemental Changes of Ferromanganese Nodules

The elemental composition of the ferromanganese nodule samples is shown in Table 1.

The outer crust of ferromanganese nodules is usually composed of ferromanganese minerals. Fe and Mn are essential metal elements. Ni and Cu are also significant elements in ferromanganese nodules, since they generally exist in ferromanganese minerals in the form of isomorphic substitution and coexist with Fe and Mn oxide aggregates. The changes of these metal elements reflect the changes in the mineral composition of ferromanganese nodules to a certain extent.

**Figure 3:**
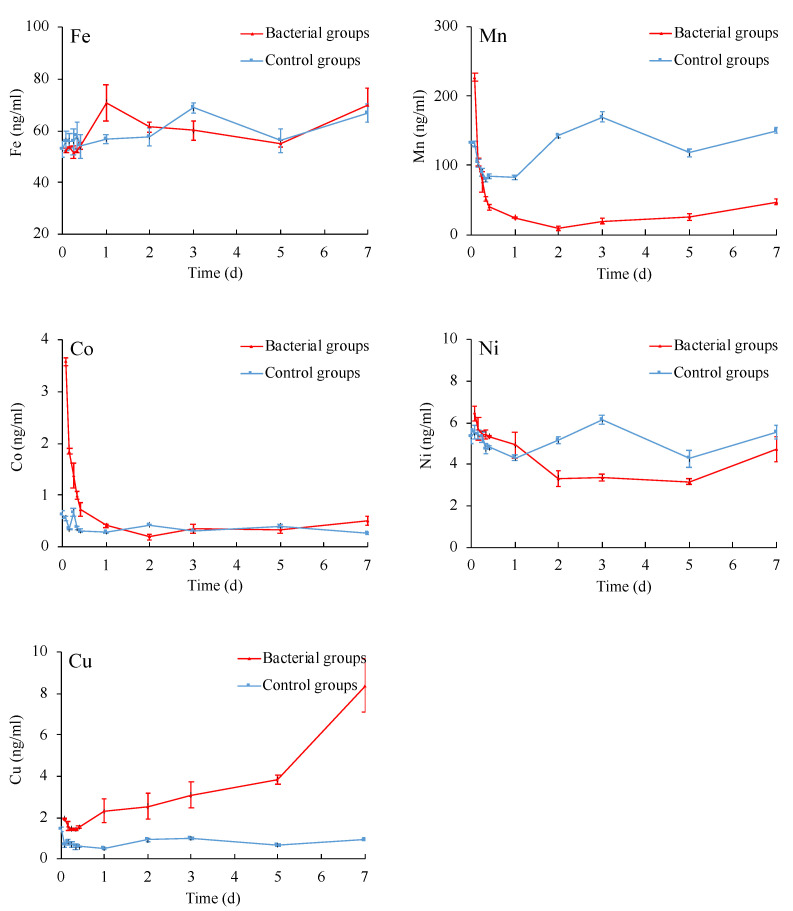
Time dependence of ion concentrations in bacterial and control groups.

The total ion concentrations of Fe, Mn, Co, Ni, and Cu in the bacterial and control groups’ reaction solution were measured for 7 days of interaction experiments (Figure 3). The Wilcoxon signed-rank test results between bacterial and control groups were shown in Table 2. Combined with the results of ion concentration and statistical test, the changes of ion concentration were speculated as follows:

Fe: The total ion concentration of Fe in the bacterial groups increased within one day and then decreased. The ion concentration in the bacterial groups approached that in the control groups after one day. Probably because strain CW126-A03 promoted the dissolution and reduction of Fe (II)(III) in ferromanganese nodules powder, the concentration of iron ions in solutions increased. Meanwhile, iron ions’ concentrations decreased due to the accumulation and precipitation effect by strain CW126-A03. Overall, the dissolution and reduction effect of Fe approached the accumulation and precipitation.

Mn: The total ion concentration of Mn in the bacterial groups increased rapidly and then decreased within one day. The ion concentration in the bacterial groups was lower than that in the control groups after one day. Probably because strain CW126-A03 promoted the dissolution and reduction of Mn (II)(IV) in ferromanganese nodules powder; thus, the concentration of manganese ions in solutions increased rapidly in a short time. Meanwhile, manganese ions concentration decreased rapidly due to the accumulation and precipitation effect by strain CW126-A03. Overall, the dissolution and reduction effect of Mn is less than the accumulation and precipitation. Therefore, a large number of dissolved manganese ions were accumulated and precipitated, resulted in the rapid decline of manganese ions concentration, which was lower than that in the control groups.

Co: The total ion concentration of Co in the bacterial groups increased rapidly and then decreased within one day. The ion concentration in the bacterial groups approached that in the control groups after one day—probably because strain CW126-A03 promoted the dissolution and reduction of Co in ferromanganese nodule powder; thus, the concentration of cobalt ions in solutions increased rapidly in a short time. Meanwhile, cobalt ions in solutions decreased rapidly due to the accumulation and precipitation effect by strain CW126-A03. Overall, the dissolution and reduction effect of Co approached the accumulation and precipitation.

Ni: The total ion concentration of Ni in the bacterial groups decreased continuously, which was lower than that in the control groups after one day—probably because the dissolution and reduction effect of Ni is less than the accumulation and precipitation, so a large number of dissolved nickel ions were accumulated and precipitated, resulting in the continuous decline of nickel ion concentration, which was lower than that in the control groups.

Cu: The total ion concentration of Cu in the bacterial groups increased continuously, which was higher than that in the control groups after one day. This is probably because strain CW126-A03 promoted the dissolution and reduction of Cu in ferromanganese nodule powder; thus, the concentration of copper ions in solutions increased continuously. Overall, the dissolution and reduction effect of Cu was greater than the accumulation and precipitation. Therefore, the copper ions concentration increased continuously and much greater than that of the control groups.

By summarizing the changes of main ions concentration in the bacterial and control groups, it can be seen that the dissolution and reduction of Fe by strain CW126-A03 were strong, the accumulation and precipitation were strong, and the dissolution and reduction effect approached the accumulation and precipitation. The dissolution and reduction of Mn were strong, the accumulation and precipitation were strong, and the dissolution and reduction effect was less than the accumulation and precipitation. The dissolution and reduction of Co were strong, the accumulation and precipitation were strong, and the dissolution and reduction effect approached the accumulation and precipitation. The dissolution and reduction of Ni were weak, the accumulation and precipitation were strong, and the dissolution and reduction effect was less than the accumulation and precipitation. The dissolution and reduction of Cu were strong, the accumulation and precipitation were weak, and the dissolution and reduction effect was greater than the accumulation and precipitation.

### 3.3. TEM Observation and Analysis of the Cells

**Figure 4:**
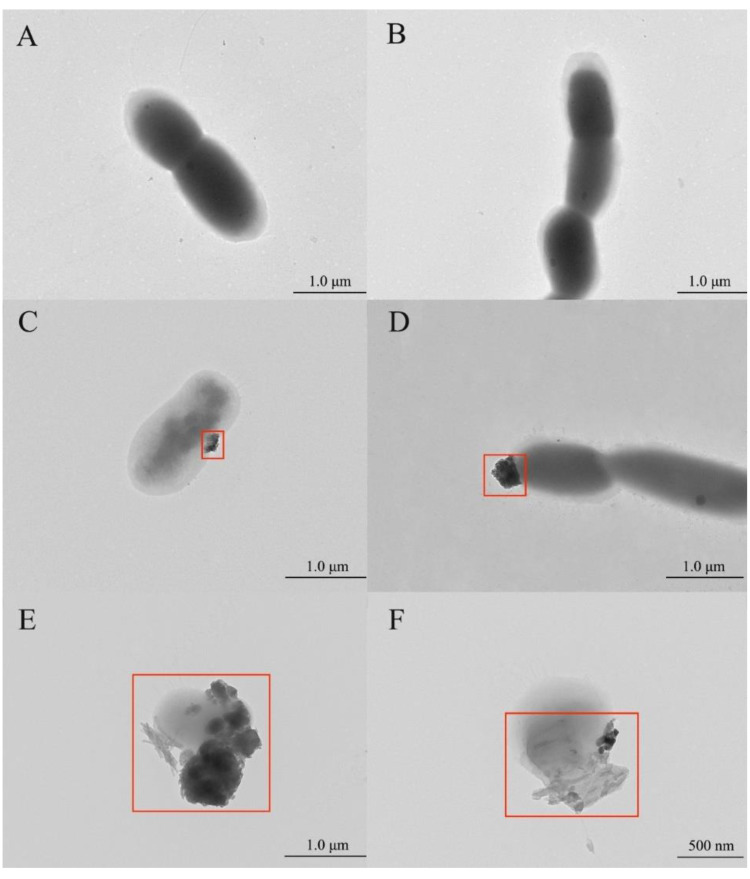
Morphology of strain CW126-A03 cells. (**A**,**B**), cells morphology cultured in growth medium; (**C**,**D**), cells morphology on the 1st day of interaction experiments; (**E**,**F**), cells morphology on the 4th day of interaction experiments.

The morphology of strain CW126-A03 was observed by TEM (Hitachi, HT7700). The cells were about an elliptical rod (0.6–1.0 × 0.9–1.6 μm, Figure 4A,B). The mineral particles formed on the cell’s surface in the bacterial groups were observed on the first and fourth day during the interaction experiments. Figure 4C,D constitutes different cell images observed on the 1st day. There were ultra-micro-sized mineral particles on the cell surface, which were about 0.2–0.3 μm in size (shown in the red box in the figure). Figure 4E,F shows different cell images observed on the 4th day. The size of mineral particles on the cell surface increased to about 1 μm (shown in the red box in the figure), embedded in the cell, and had a crystal shape.

The results showed that there were no mineral particles on the surface of cells cultured in the growth medium. In the interaction experiments, ultra-micro-sized mineral particles were formed on the cells’ surface. These observations implied the biomineralization process of strain CW126-A03. With the increase of reaction time, the size of mineral particles increased, and crystalline minerals formed.

**Figure 5:**
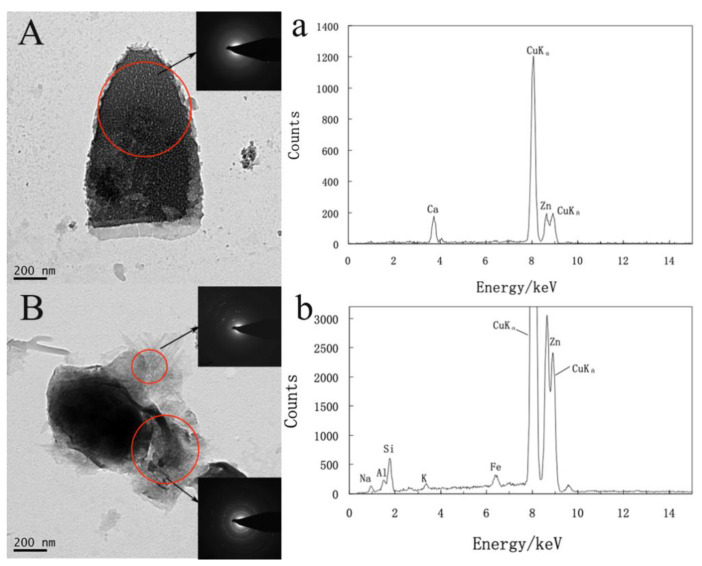
TEM images with SAED patterns of minerals formed on bacteria cells’ surface (**A**,**B**); the EDS spectrum of minerals (**a**,**b**). Cu and Zn are background signals in the EDS spectrum.

TEM images with the selected area electron diffraction (SAED) patterns were used to analyze and identify formed ultra-micro-sized mineral particles (Figure 5). The multi-point determination of multiple samples and the same sample was carried out. The results of element composition in energy dispersive spectroscopy (EDS) are similar, the element strength is slightly different. The early formed nano-precipitates were amorphous, and the EDS spectrum showed that Ca content was high, as shown in Figure 5A(a). They transformed to crystalline minerals with time changes, and the EDS spectrum showed the elemental composition of the minerals, including Na, Al, Si, K, and Fe (Figure 5B(b)). According to the electron diffraction patterns analysis of the mineral particles, the speciation of the minerals showed a polycrystalline phase. Combined with the EDS analysis, it may be silicate minerals containing Fe. Some minerals with good crystallinity showed a strong bright spot in the electron diffraction figure. Based on the d-spacing analysis of the electron diffraction patterns, we speculated that there were iron-containing silicate minerals, such as Pyroxene [Ca (Mg, Fe, Al) (Si, Al)_2_O_6_], Esseneite (CaFeAlSiO_6_), Clinopyroxene [(Ca, Mg, Fe)_2_Si_2_O_6_].

### 3.4. XRD Analysis of Ferromanganese Nodules Mineral Composition

**Figure 6:**
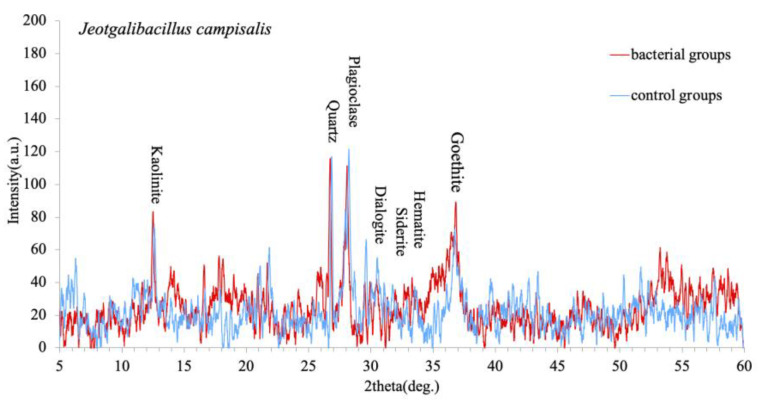
Mineral composition changes of ferromanganese nodules crust.

After the interaction experiments, the ferromanganese nodule powder in the solution was collected by centrifugation, and the mineral composition of the nodules was analyzed by XRD, as shown in Figure 6. The goethite content increased slightly in the interaction with *J. campisalis* CW126-A03, which may be related to biomineralization. Bacteria can form the amorphous iron hydroxide or oxide, such as ferrihydrite, in the iron-containing solution. The ferrihydrite must be converted into stable minerals such as hematite or goethite. Although hematite is a relatively stable mineral, the goetherization of hematite still exists in nature. Iron exists in Fe^2+^ firstly, then oxidizes to Fe^3+^, forms goethite or jarosite, and then converts to FeOOH. The reaction formula is Fe_2_O_3_ + H_2_O = 2Fe (OOH) [36]. Combined with the changes in ion concentrations in solution and TEM results, we speculated that there is biomineralization in the interaction experiments.

## 4. Discussion

### 4.1. The Mechanism of Microbial-Ferromanganese Nodule Interaction

Mineral nucleation involves the spontaneous growth of many nuclei that are large enough to resist rapid dissolution. The formation of these ‘critical nuclei’ requires a certain degree of supersaturation, in which the ion concentration in solution exceeds the solubility product of the mineral phase [37]. After the formation of critical nuclei, the continuous addition of ions is accompanied by the decrease of free energy, leading to the growth of minerals. This process is spontaneous until the system reaches equilibrium. However, a certain amount of energy is needed to form a new interface between the potential mineral nucleus and the aqueous solution. The required energy is an activation energy barrier. Microorganisms can reduce the activation energy barrier and promote mineral nucleation and growth. The enzymes secreted by microorganisms are highly catalytic, reducing the Gibbs free energy of some thermodynamic reactions and overcoming the kinetic obstacles of chemical reactions [38]. Microorganisms can also reduce the free energy by the cell surface abundant functional groups [39].

The microbial-ferromanganese nodule interaction in this paper comprises two processes: mineral dissolution promoted by microorganisms and biomineralization.

Microorganisms can decompose almost all types of minerals and rocks and release various mineral elements [40]. Microorganisms extract nutrients by attaching and dissolving minerals with the effect of produced surface polymers, enzymes and siderophores [41,42]. The critical properties of siderophores include dissolving iron minerals, supporting bacterial growth, and decreasing the Gibbs energy of iron oxide dissolution reactions. Siderophores can dissolve several manganese oxides via reduction and nonreduction pathways to form siderophore-Mn (III) complexes in several aquatic environments [43,44]. The growth curve of strain CW126-A03 (Figure 2) and the ion concentration curves (Figure 3) were compared and analyzed. Within 2 h of the interaction experiments, the growth curve of strain CW126-A03 was in the lag phase, and the cells were few, and the ion concentration of elements in bacterial and control groups has not changed much. Within 2–24 h of the interaction experiments, the growth curve was in the log phase, and cell number increased rapidly. Bacteria cells may attach to mineral surfaces and secrete metabolic secretions such as extracellular polymeric substances (EPS), enzymes, and siderophores to promote mineral dissolution [45,46]. On the first day of the interaction experiments, the concentration of Fe, Mn, and Co ions in the bacteria groups increased rapidly and then decreased (Figure 3), of which the strain CW126-A03 secretions may promote the release of elements. The ocean was rich in iron for a long time in the past [47], and the concentration of dissolved Fe (II) was estimated to be 0.02 mM [48,49], which was about 1000–10,000 times the modern seawater concentration. The content of Mn and Fe in modern seawater is relatively low; the average concentration of Mn ranges from 0.02 to 10 μg/L [50], whereas the average concentration of Fe is approximately 3 μg/L [51]. The maximum Fe and Mn concentrations in our bacterial groups were about 70 μg/L and 210 μg/L, respectively (Figure 3). Although this value cannot be compared with ancient seawater, it was still much higher than modern seawater. The ion supersaturation’s critical nuclei may occur, followed by decreased ion concentration in the bacteria groups. Generally, the amorphous solid phase is easy to form in mineral growth because of its high degree of hydration, high solubility, and lack of intrinsic form [52]. If the solution composition exceeds its solubility, minerals such as ferric hydroxide [Fe (OH)_3_] will easily nucleate. In most natural systems, ferric hydroxide is a more stable precursor of iron oxides, such as goethite (FeOOH) and hematite (Fe_2_O_3_). The ferric hydroxide associated with the microbial surface can be transformed to the iron oxide encrusted in the cell surface [53]. The increase of goethite content in XRD results (Figure 6) may be related to this mechanism. The formation of iron oxide needs higher interfacial free energy, of which the bacteria may contribute significantly to the mineral precipitation by biomineralization.

The bacterial cell wall is commonly overlaid by additional organic layers, such as EPS, sheaths, and S-layers, which differ in hydration, composition, and structure [54]. The bacterial cell surface acts as a highly reactive interface and provides a nucleation site for mineral precipitation [55]. The cell surface has organic ligands, such as carboxyl, hydroxyl, amine, and phosphate functional groups. Most of these organic ligands can deprotonate, making the cell surface negatively charged [53] and thus become reactive towards charged cations [56,57]. Some cations preferentially bind to different sites on the cell surface, such as trivalent and divalent metal cations that are firmly bound to the cell wall of various bacteria. Subsequently, the interfacial energy of solid phase heterogeneous nucleation decreases, and the surface area of the nucleus in contact with the bulk solution, decreases. These cations react with more ions and may lead to mineral precipitation. Bacteria promote the precipitation of minerals on the cell surface [58] and form microbial-mineral complexes. The mineralization goes through a series of stages, usually from the adsorption to EPS or wall material, followed by the nucleation of small (<100 nm in diameter) grains, and with sufficient time, the complete encrustation of the cell [13]. In the interaction experiments, metal ions such as Fe, Mn, Co, and Ni in bacterial groups appeared to precipitate on the surface of bacterial cells to form ultra-micro-sized mineral particles (Figure 4C,D). With time increase, newly crystalline minerals gradually formed (Figure 4E,F). Due to the high ion concentrations of the biophilic elements such as Fe and Mn in the reaction solution, they are easier to be biomineralized by strain CW126-A03 to form Fe-containing or Mn-containing minerals. The possible existence of Fe-containing silicate minerals, such as pyroxene, esseneite, and clinopyroxene, is shown in TEM results (Figure 5). The bacterial wall contains a large amount of mineral precipitate by biomineralization, and the cell becomes the core of the newly formed mineral [24].

### 4.2. Biomineralization Promote the Formation of Ferromanganese Nodules

Microorganisms play an important role in the precipitation of iron and manganese in marine sediments and may accelerate the formation of ferromanganese nodules. Much of the previous work focused on the BCM effect in nodules formation, but data for BIM were largely not considered. We conducted the interaction experiments to verify the possible BIM effect of microbial cell characteristics in the formation of ferromanganese nodules. Non-ferromanganese nodules and non-MnOB bacterium were selected as experimental strain; the results indicated that the microbial-ferromanganese nodule interaction was the process of dissolution and re-mineralization. The bacterial cells may act as templates to induce nodules’ mineral precipitation when the ion concentration in the surrounding environment was over-saturated. Wang et al. succeeded in identifying microorganisms covered with S-layers in the nodules, increasing the possibility of forming ferromanganese nodules by BIM [59]. The ion concentration of Fe and Mn in the ocean was very high, and the supersaturation is more likely to occur to form the critical nucleus, and the marine microorganisms may promote the deposition and formation of ferromanganese nodules’ mineral through biomineralization. Combined with the total biomass of marine microorganisms, biomineralization may play an important role in distributing metal elements from the hydrosphere to the marine sediment. For example, the extensive records of banded iron formation (BIF) from 3.8 to 0.5 billion years ago demonstrated the enormous magnitude of ferric iron deposition to the seafloor. Indirect evidence suggested that microbial activity was involved in the initial deposition of iron deposits, followed by consolidation to form BIF [60,61,62]. We speculated that the BIM of microorganisms, i.e., bacteria, may promote the formation of marine ferromanganese nodules.

## 5. Conclusions

The microbial-ferromanganese nodule interaction is as follows: microorganisms promote the dissolution of ferromanganese nodules via secretions produced, and release major elements such as Fe and Mn; the cell surface as a nucleation site of minerals precipitation when the ion concentration over-saturated. The biomineralization process begins from ions adsorption on the cell wall, then to tiny mineral grains nucleation, and finally to complete encrustation. The BIM appeared to promote the formation of ferromanganese nodules’ minerals. This BIM hypothesis provides another perspective for the further understanding of the formation of ferromanganese nodules in the deep ocean, and may be of great significance for future nodule exploration.

## Figures and Tables

**Table 1:** Elemental composition of the crust powder.

Element	Si	Fe	Al	Ca	Mg	Na	K	Mn	Cl	S
Content (PPM)	194,180	202,440	24,122.4	30,773.4	9120	8904	5808.6	104,174.6	132	229.1
Element	Ti	P	Ni	Co	Ba	Pb	Ce	Sr	Cu	V
Content (PPM)	10,483.9	4431.9	2117.8	2125.4	321	1277.7	1285.3	1098	1285.3	764.2
Element	Zr	Zn	As	Nd	La	Mo	Y	Cr	W	Nb
Content (PPM)	541	496.8	489.6	445.9	321	297	160.6	83	67.2	45.6
Element	Th	Sb	Hf	Cd	Bi	Rb	Sc	Ga	U	Br
Content (PPM)	42.1	12	14.8	—	8.4	7.2	4.5	2.7	0.1	0

**Table 2:** The Wilcoxon signed-rank test results between bacterial and control groups.

Fe	Mn	Co	Ni	Cu
0.4922 ^ns^	0.04883 *	0.03654 *	0.625 ^ns^	0.001953 *

(* for *p* < 0.05; ns for *p* > 0.05).

## Data Availability

The data presented in this study are available on request from the corresponding author.

## References

[1] Hein J.R., Koschinsky A. (2014). Deep-Ocean Ferromanganese Crusts and Nodules. Treatise on Geochemistry.

[2] Hein J.R., Harff J., Meschede M., Petersen S., Thiede J. (2016). Manganese Nodules. Encyclopedia of Marine Geosciences.

[3] Hein J.R., Mizell K., Koschinsky A., Conrad T.A. (2013). Deep-Ocean Mineral Deposits As a Source of Critical Metals for High—and Green-Technology Applications: Comparison With Land-Based Resources. Ore Geol. Rev..

[4] Verlaan P.A., Cronan D.S., Morgan C.L. (2004). A comparative analysis of compositional variations in and between marine ferromanganese nodules and crusts in the South Pacific and their environmental controls. Prog. Oceanogr..

[5] Koschinsky A., Halbach P. (1995). Sequential leaching of marine ferromanganese precipitates: Genetic implications. Geochim. Cosmochim. Acta.

[6] Benites M., Millo C., Hein J., Nath B.N., Murton B., Galante D., Jovane L. (2018). Integrated geochemical and morphological data provide insights into the genesis of ferromanganese nodules. Minerals.

[7] Thiel G.A. (1925). Manganese precipitated by micro-organisms. Econ. Geol..

[8] Shulse C.N., Maillot B., Smith C.R., Church M.J. (2017). Polymetallic nodules, sediments, and deep waters in the equatorial North Pacific exhibit highly diverse and distinct bacterial, archaeal, and microeukaryotic communities. MicrobiologyOpen.

[9] Ehrlich H.L. (1996). How microbes influence mineral growth and dissolution. Chem. Geol..

[10] Xie S., Liu D., Qiu X., Huang X., Algeo T.J. (2016). Microbial roles equivalent to geological agents of high temperature and pressure in deep Earth. Sci. China Earth Sci..

[11] Douglas S., Beveridge T.J. (1998). Mineral formation by bacteria in natural microbial communities. FEMS Microbiol. Ecol..

[12] Jiang M., Ohnuki T., Kozai N., Tanaka K., Suzuki Y., Sakamoto F., Kamiishi E., Utsunomiya S. (2010). Biological nano-mineralization of Ce phosphate by Saccharomyces cerevisiae. Chem. Geol..

[13] Jiang M., Ohnuki T., Tanaka K., Kozai N., Kamiishi E., Utsunomiya S. (2012). Post-adsorption process of Yb phosphate nano-particle formation by Saccharomyces cerevisiae. Geochim. Cosmochim. Acta.

[14] Jiang M., Ohnuki T., Utsunomiya S. (2018). Biomineralization of Middle Rare Earth Element Samarium in Yeast and Bacteria Systems. Geomicrobiol. J..

[15] Ohnuki T., Jiang M., Sakamoto F., Kozai N., Yamasaki S., Yu Q., Tanaka K., Utsunomiya S., Xia X., Yang K. (2015). Sorption of trivalent cerium by a mixture of microbial cells and manganese oxides: Effect of microbial cells on the oxidation of trivalent cerium. Geochim. Cosmochim. Acta.

[16] Braissant O., Decho A.W., Dupraz C., Glunk C., Przekop K.M., Visscher P.T. (2007). Exopolymeric substances of sulfate-reducing bacteria: Interactions with calcium at alkaline pH and implication for formation of carbonate minerals. Geobiology.

[17] Bazylinski D.A., Frankel R.B. (2000). Biologically controlled mineralization of magnetic iron minerals by magnetotactic bacteria. Environ. Microbe-Met. Interact..

[18] Rodelli D., Jovane L., Roberts A.P., Cypriano J., Abreu F., Lins U. (2018). Fingerprints of partial oxidation of biogenic magnetite from cultivated and natural marine magnetotactic bacteria using synchrotron radiation. Environ. Microbiol. Rep..

[19] Hassan M.B., Rodelli D., Benites M., Abreu F., Murton B., Jovane L. (2020). Presence of biogenic magnetite in ferromanganese nodules. Environ. Microbiol. Rep..

[20] Nitahara S., Kato S., Usui A., Urabe T., Suzuki K., Yamagishi A. (2017). Archaeal and bacterial communities in deep-sea hydrogenetic ferromanganese crusts on old seamounts of the northwestern Pacific. PLoS ONE.

[21] Rosson R.A., Nealson K.H. (1982). Manganese binding and oxidation by spores of a marine bacillus. J. Bacteriol..

[22] Gutteridge S., Tanner S., Bray R. (1978). The molybdenum centre of native xanthine oxidase. Evidence for proton transfer from substrates to the centre and for existence of an anion-binding site. Biochem. J..

[23] Larock P.A., Ehrlich H.L. (1975). Observations of bacterial microcolonies on the surface of ferromanganese nodules from Blake Plateau by scanning electron microscopy. Microb. Ecol..

[24] Ehrlich H.L. (1998). Geomicrobiology: Its significance for geology. Earth-Sci. Rev..

[25] Melton E.D., Swanner E.D., Behrens S., Schmidt C., Kappler A. (2014). The interplay of microbially mediated and abiotic reactions in the biogeochemical Fe cycle. Nat. Rev. Microbiol..

[26] Konhauser K.O., Kappler A., Roden E.E. (2011). Iron in microbial metabolisms. Elements.

[27] Shiraishi F., Mitsunobu S., Suzuki K., Hoshino T., Morono Y., Inagaki F. (2016). Dense microbial community on a ferromanganese nodule from the ultra-oligotrophic South Pacific Gyre: Implications for biogeochemical cycles. Earth Planet. Sci. Lett..

[28] Tully B.J., Heidelberg J.F. (2013). Microbial communities associated with ferromanganese nodules and the surrounding sediments. Front. Microbiol..

[29] Blöthe M., Wegorzewski A., Müller C., Simon F., Kuhn T., Schippers A. (2015). Manganese-cycling microbial communities inside deep-sea manganese nodules. Environ. Sci. Technol..

[30] Lindh M.V., Maillot B.M., Shulse C.N., Gooday A.J., Amon D.J., Smith C.R., Church M.J. (2017). From the surface to the deep-sea: Bacterial distributions across polymetallic nodule fields in the clarion-clipperton zone of the Pacific Ocean. Front. Microbiol..

[31] Nayak B., Das S.K., Munda P. (2013). Biogenic signature and ultra microfossils in ferromanganese nodules of the Central Indian Ocean Basin. J. Asian Earth Sci..

[32] Jiang X.-D., Sun X.-M., Guan Y. (2019). Biogenic mineralization in the ferromanganese nodules and crusts from the South China Sea. J. Asian Earth Sci..

[33] Yoon J.-H., Kang S.-J., Schumann P., Oh T.-K. (2010). Jeotgalibacillus salarius sp. nov., isolated from a marine saltern, and reclassification of Marinibacillus marinus and Marinibacillus campisalis as Jeotgalibacillus marinus comb. nov. and Jeotgalibacillus campisalis comb. nov., respectively. Int. J. Syst. Evol. Microbiol..

[34] Yoon J.-H., Kim I.-G., Schumann P., Oh T.-K., Park Y.-H. (2004). Marinibacillus campisalis sp. nov., a moderate halophile isolated from a marine solar saltern in Korea, with emended description of the genus Marinibacillus. Int. J. Syst. Evol. Microbiol..

[35] Cao W.-R., Lu D.-C., Sun X.-K., Sun Y.-Y., Saren G., Yu X.-K., Du Z.-J. (2020). Seonamhaeicola maritimus sp. nov., isolated from coastal sediment. Int. J. Syst. Evol. Microbiol..

[36] Schwertmann U. (1971). Transformation of hematite to goethite in soils. Nature.

[37] Stumm W., Morgan J. (1996). Aquatic Chemistry, Chemical Equilibria and Rates in Natural Waters.

[38] Albery W.J., Knowles J.R. (1976). Evolution of enzyme function and the development of catalytic efficiency. Biochemistry.

[39] Ferris F. (2000). Microbe-Metal Interactions in Sediments. Microbial Sediments.

[40] Baker M., Lalonde S., Konhauser K., Foght J. (2010). Role of extracellular polymeric substances in the surface chemical reactivity of Hymenobacter aerophilus, a psychrotolerant bacterium. Appl. Environ. Microbiol..

[41] Roberts J.A., Fowle D.A., Hughes B.T., Kulczycki E. (2006). Attachment behavior of Shewanella putrefaciens onto magnetite under aerobic and anaerobic conditions. Geomicrobiol. J..

[42] Maurice P.A., Haack E.A., Mishra B. (2009). Siderophore sorption to clays. Biometals.

[43] Duckworth O.W., Sposito G. (2007). Siderophore-promoted dissolution of synthetic and biogenic layer-type Mn oxides. Chem. Geol..

[44] Peña J., Duckworth O.W., Bargar J.R., Sposito G. (2007). Dissolution of hausmannite (Mn3O4) in the presence of the trihydroxamate siderophore desferrioxamine B. Geochim. Cosmochim. Acta.

[45] Welch S., Barker W., Banfield J. (1999). Microbial extracellular polysaccharides and plagioclase dissolution. Geochim. Cosmochim. Acta.

[46] Barker W., Welch S., Chu S., Banfield J. (1998). Experimental observations of the effects of bacteria on aluminosilicate weathering. Am. Mineral..

[47] Kendall B., Anbar A.D., Kappler A., Konhauser K.O. (2012). The global iron cycle. Fundam. Geobiol..

[48] Holland H.D. (1973). The oceans; a possible source of iron in iron-formations. Econ. Geol..

[49] Morris R. (1993). Genetic modelling for banded iron-formation of the Hamersley Group, Pilbara Craton, Western Australia. Precambrian Res..

[50] Xiao-quan S., Radziuk B., Welz B., Vyskočilová O. (1993). Determination of manganese in river and sea-water samples by electrothermal atomic absorption spectrometry with a tungsten atomizer. J. Anal. At. Spectrom..

[51] Anthoni J.F. (2006). The chemical composition of seawater. Magnesium.

[52] Nielsen A.E., Söhnel O. (1971). Interfacial tensions electrolyte crystal-aqueous solution, from nucleation data. J. Cryst. Growth.

[53] Konhauser K.O. (2009). Introduction to Geomicrobiology.

[54] Konhauser K., Riding R. (2012). Bacterial biomineralization. Fundamentals of Geobiology.

[55] Dove P., De Yoreo J., Weiner S. (2003). Biomineralization.

[56] Beveridge T., Murray R. (1976). Uptake and retention of metals by cell walls of Bacillus subtilis. J. Bacteriol..

[57] Beveridge T., Murray R. (1980). Sites of metal deposition in the cell wall of Bacillus subtilis. J. Bacteriol..

[58] Kashefi K., Lovley D.R. (2000). Reduction of Fe (III), Mn (IV), and toxic metals at 100 °C by Pyrobaculum islandicum. Appl. Environ. Microbiol..

[59] Wang X.-H., Gan L., Müller W.E. (2009). Contribution of biomineralization during growth of polymetallic nodules and ferromanganese crusts from the Pacific Ocean. Front. Mater. Sci. China.

[60] Konhauser K.O., Hamade T., Raiswell R., Morris R.C., Ferris F.G., Southam G., Canfield D.E. (2002). Could bacteria have formed the Precambrian banded iron formations?. Geology.

[61] Kappler A., Pasquero C., Konhauser K.O., Newman D.K. (2005). Deposition of banded iron formations by anoxygenic phototrophic Fe (II)-oxidizing bacteria. Geology.

[62] Posth N.R., Hegler F., Konhauser K.O., Kappler A. (2008). Alternating Si and Fe deposition caused by temperature fluctuations in Precambrian oceans. Nat. Geosci..

